# Deferred versus Expedited Aortic Valve Replacement in Patients with Symptomatic Severe Aortic Stenosis During the SARS-CoV-2 Pandemic (AS DEFER): A Research Letter

**DOI:** 10.5334/gh.989

**Published:** 2021-04-30

**Authors:** Jonas Lanz, Christoph Ryffel, Noé Corpataux, Nicole Reusser, Taishi Okuno, Bettina Langhammer, David Reineke, Fabien Praz, Stefan Stortecky, Stephan Windecker, Thomas Pilgrim

**Affiliations:** 1 Department of Cardiology, Inselspital, Bern University Hospital, University of Bern, CH; 2 Department of Cardiovascular Surgery, Inselspital, Bern University Hospital, University of Bern, CH

**Keywords:** COVID-19, aortic valve stenosis, aortic valve replacement, transcatheter aortic valve implantation

The Swiss Federal Council banned elective interventions in all hospitals in Switzerland during the SARS-CoV-2 pandemic between March 20 and April 26, 2020 [1]. A triage algorithm was prospectively implemented to allocate patients with symptomatic severe aortic stenosis to expedited versus deferred aortic valve replacement (AVR). A preliminary evaluation of our algorithm has been reported previously and focused on clinical events during the wait time for AVR in the deferred treatment arm [2]. Here, we report the pre-specified primary endpoint results at six months. In contrast to the preliminary report, the present analysis reflects not only the events during the wait time but also the events associated with delayed AVR in the deferred treatment arm.

**Figure 1:**
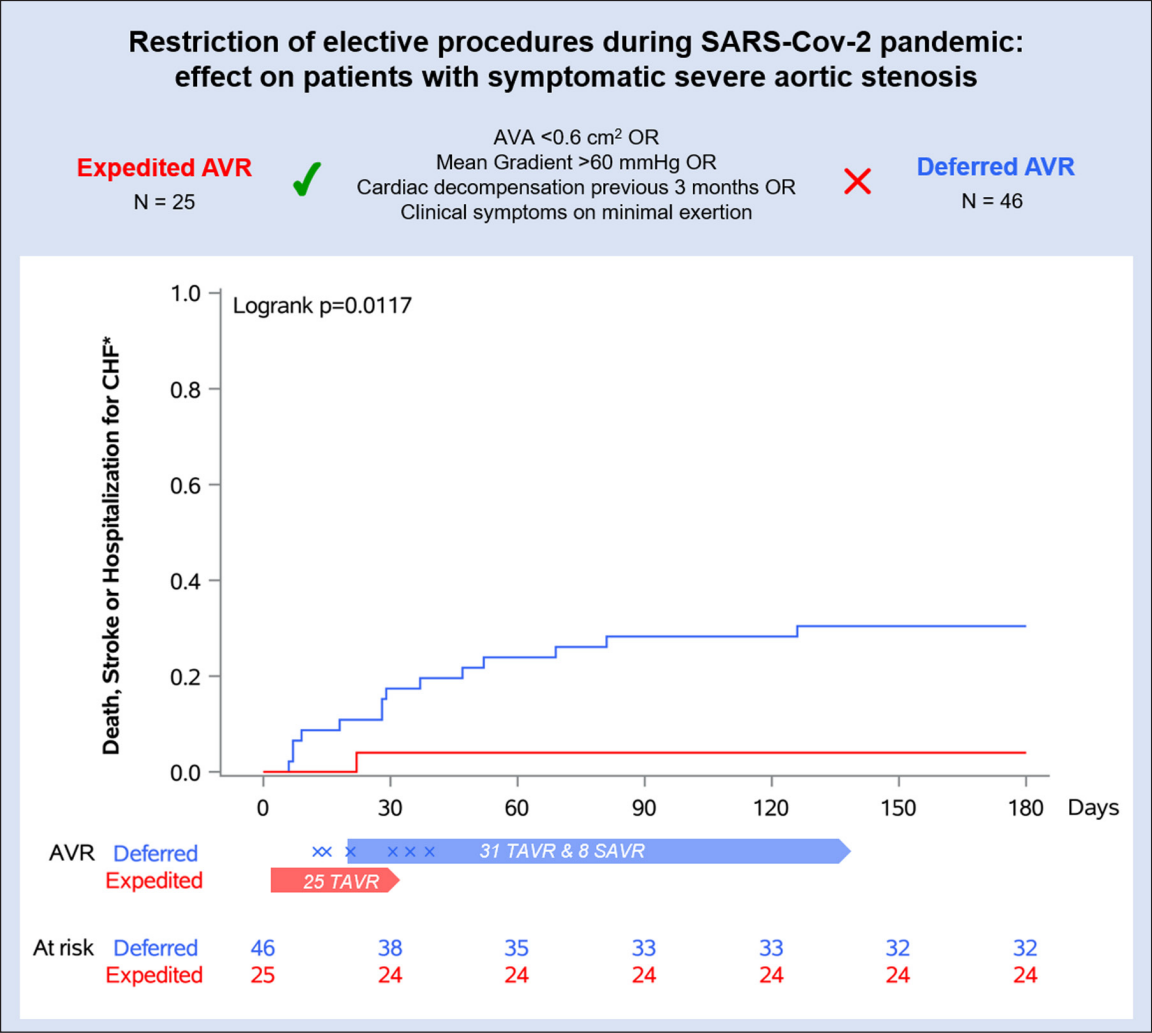
Pre-specified treatment strategy allocation algorithm and cumulative event curves for the primary composite endpoint of all-cause death, stroke and hospitalization for congestive heart failure (CHF). The crosses mark the time points of aortic valve replacement (AVR) in patients who crossed over to expedited AVR.

The AS DEFER study is a prospective cohort study of patients with symptomatic severe aortic stenosis referred for AVR during the SARS-CoV-2-related ban of elective procedures in Switzerland. Severe aortic stenosis was defined by an aortic valve area (AVA) ≤1.0 cm^2^ or <0.6 cm^2^/m^2^. Patients with critical aortic stenosis defined by an AVA of ≤0.6 cm^2^, a transvalvular mean gradient of ≥60 mmHg, a history of cardiac decompensation during the previous three months or clinical symptoms on minimal exertion underwent expedited AVR (Figure 1). Patients with stable symptoms were scheduled for deferred AVR. Instruments of data collection and follow-up have been detailed previously [2]. The primary endpoint was a composite of all-cause mortality, stroke, and hospitalization for heart failure by intention-to-treat as assessed at six months.

The study was approved by the local ethics committee and registered with ClinicalTrials.gov (NCT04333875). All patients provided informed consent for participation in this study. Cumulative event curves were generated based on the Kaplan Meier method and compared using the Log rank test. Hazard ratios were calculated by means of Cox proportional hazards regression with adjustment for age and STS-PROM score; the proportionality assumption was tested by including time-dependent covariates.

Between March 20 and April 26, 2020, a total of 82 individuals were referred for AVR and were assessed for eligibility. After exclusion of 11 subjects, 71 patients (45% female, STS-PROM 3.1 ± 2.4) with symptomatic severe aortic stenosis and a mean age of 78.0 ± 7.5 years were prospectively enrolled into the study. Twenty-five patients (35.2%) fulfilling the criteria for critical AS underwent expedited AVR according to the pre-specified algorithm; AVR was deferred in 46 patients (64.8%). The median interval between treatment allocation and AVR was seven days (IQR 2 to 17) in the expedited and 55 days (IQR 36 to 80) in the deferred group (p < 0.0001). Baseline characteristics of patients have been reported previously [2]. Clinical follow-up at six months was complete in all patients. A total of 35 (49.3%) patients were tested for SARS-CoV-2; two (5.7%) of them were positive. At six months, the primary endpoint occurred in one (4%) patient in the expedited and in 14 (30%) patients in the deferred group (log rank p = 0.0117; adjusted hazard ratio: 0.12 (95%-CI: 0.01 to 0.60)) (Figure 1). Two patients (4.3%) in the deferred group died, none of them from SARS-CoV-2. One patient (4.0%) in the expedited and three (6.5%) in the deferred group suffered a stroke. Ten patients (21.7%) in the deferred group were hospitalized for heart failure; seven crossed over to expedited AVR. Periprocedural events were limited to one stroke (4.0%) in the expedited and two (4.3%) in the deferred arm. Beyond the peri-procedural phase after AVR, two patients in the deferred group experienced an adverse event (6.5%) (one death, one heart failure hospitalization); conversely, none of the patients in the expedited group experienced an event during this period after AVR.

In this prospective cohort study of patients with severe symptomatic aortic stenosis, deferred AVR was associated with an increased risk of the primary composite of all-cause mortality, stroke, and hospitalization for heart failure. Increased mortality and heart failure hospitalizations due to wait time have recently been reported for severe aortic stenosis patients in a retrospective cohort study in Ontario [3]. Our data suggest that also patients with non-critical aortic stenosis referred for AVR are at substantial risk of adverse outcomes if treatment is deferred. The risk may not be limited to the wait time for intervention, but may also carry on to late outcome after AVR.

Study limitations are as follows: First, the number of patients in both treatment arms were modest and event rates preclude a multivariable assessment of predictors for clinical endpoints. Second, the number of SARS-CoV-2 patients did not exceed available hospital resources and the duration of the ban of elective procedures was limited to 38 days; hence, the effect of treatment deferral may be even more accentuated with overstrained resources or a long-lasting ban.

In conclusion, patients with symptomatic severe aortic stenosis referred for treatment should undergo timely AVR as deferral is associated with adverse clinical outcomes.
